# Early Energy Intake and Amino Acid Profile in Preterm Newborns: A Quasi-Experimental Study

**DOI:** 10.3390/nu15132917

**Published:** 2023-06-27

**Authors:** Giovanni Boscarino, Claudia Carducci, Maria Giulia Conti, Maria Podagrosi, Annamaria Gigliello, Maria Di Chiara, Monica Bartolucci, Roberto Brunelli, Pasquale Parisi, Antonio Angeloni, Gianluca Terrin

**Affiliations:** 1 Department of Maternal and Child Health, Policlinico Umberto I, Sapienza University of Rome, 00161 Rome, Italyroberto.brunelli@uniroma1.it (R.B.); 2 Department of Experimental Medicine, Policlinico Umberto I, Sapienza University of Rome, 00185 Rome, Italy; 3 Department of Neuroscience, Mental Health and Sense Organs (NESMOS), Faculty of Medicine and Psychology, Sant Andrea Hospital University, Sapienza University of Rome, 00189 Rome, Italy

**Keywords:** protein intake, nutrition, nutritional intake, parenteral nutrition, proline, tyrosine, leucine, isoleucine, very low birth weight, plasma amino acid, neonate

## Abstract

(1) Background: An increased protein intake via parenteral nutrition (PN) in early life is associated with an improvement of the nitrogen balance in preterm newborns. However, the role of energy intake on amino acid (AA) utilization provided by PN remains to be defined. We investigated the effects of energy intake on blood AA levels and profiles. (2) Methods: Quasi-experimental study including preterm very low birth weight newborns who received an energy enhanced PN (Cohort A) or an energy standard PN (Cohort B), with a similar protein amount in the first week of life. Blood AA levels were measured between three and seven days of life (T0) and at fifteen days of life (T1) and compared between the two study cohorts. (3) Results: AA levels of 40 newborns from each group were analyzed. No difference was found for total essential and non-essential blood AA concentration at T0 between the two study cohorts. At T1, we found a significantly higher blood concentration of leucine, isoleucine and proline, and a significantly lower concentration of tyrosine in Cohort B. However, multivariate analysis did not confirm this result. (4) Conclusions: An enhanced PN protocol in terms of energy but not of protein did not influence AA levels and profiles. Considering the high risk of side effects, we suggest exercising caution when administering high energy intake via PN in the first week of life.

## 1. Introduction

Malnutrition in early life has been demonstrated to be strongly associated with stunted growth and neurological delays for infants born preterm [1,2]. Considering the immaturity of the intestinal tract, parenteral nutrition (PN) remains the preferred route to administer nutrients in preterm newborns [3]. Guidelines for PN recommend high protein and energy intake early in life for preterm newborns [2,4,5]. Studies evaluating intravenous amino acid (AA) administration during the first days of life have found an improvement in the nitrogen balance and growth of this population [6,7,8]. However, the optimal AA-energy ratio in PN is still largely undefined. If increased nutritional intake in early life is associated with an improvement in the growth of preterm newborns, a suboptimal range of the protein to energy ratio leads to adverse consequences [2,4,9,10,11]. When energy intake is inadequate, proteins can be used as an energy source, and thus the nitrogen balance becomes less positive [12,13,14]. Increasing caloric intake can prevent protein loss and improve nitrogen retention [12]. On the other hand, if there is a surfeit of energy with limited protein intake, the protein retention reaches a plateau, and a considerable amount of amino acids can be oxidized, altering the production of proteins [12,13]. This mechanism has been hypothesized in experimental studies [13,14], while for neonates, in a study population of 24 very low birth weight (VLBW) infants performed in 1981, Duffy et al. hypothesized that body protein levels and metabolism depend not only on endogenous AA, but also on energy intake given via PN at the end of the first week of life [15]. The two study populations received different energy intakes and protein qualities, suggesting that an increased energy intake improved *N*-retention by enhancing AA reutilization for protein synthesis, whereas a higher quality protein improved *N*-retention by limiting protein breakdown [15]. Thus, the role of energy intake on protein metabolism has not been investigated. More recently, studies have reported several negative effects of energy intakes in VLBW newborns [9,16,17]. In particular, early enhanced energy intake via PN increases the risk of metabolic side effects in the first week of life, such as hyperglycemia, hypertriglyceridemia, metabolic acidosis and dyselectrolytemia [9,11,16,18,19,20] and affects long-term neurodevelopment [9,17]. Beginning with these considerations, we designed a quasi-experimental study to investigate whether different energy intakes influence blood AA levels in VLBW infants.

## 2. Materials and Methods

### 2.1. Study Population and Matching

This quasi-experimental study included all newborns with gestational age (GA) less than 32 weeks or birth weight (BW) less than 1500 g consecutively admitted to the Neonatal Intensive Care Unit (NICU) of the Sapienza University Hospital “Policlinico Umberto I” in Rome, from January 2015 to December 2019, who received PN for at least seven days. Among 172 prospectively enrolled newborns in a previous study [17], we selected the first 40 newborns for this study and divided them into two cohorts. As previously described, the two cohorts of newborns received different energy intakes through PN in the first week of life [17]. We excluded newborns with major congenital malformations, inborn errors of metabolism, congenital infections, hospital discharge or transfer, or those who died within 24 h of life [21,22,23,24]. We also enrolled 3500 term breastfed neonates born during the enrollment period, who served as controls from which to derive reference value levels of AA.

### 2.2. Data Collection and Laboratory Sampling

We prospectively recorded prenatal, perinatal, and postnatal data, as previously described [17]. We also collected nutritional intake on PN and enteral nutrition (EN) daily. Diagnosis of prematurity-related morbidities was performed according to standard criteria, and defined as previously described [22,25,26,27,28].

Protocol of EN and PN intake was the same as described in previous studies [17,29]. Newborns in Cohort A received an enhanced PN energy intake during the study period, while newborns in Cohort B received a standard PN energy intake, in line with recent European Society for Pediatric Gastroenterology Hepatology and Nutrition guidelines [2,4]. The enteral feeding protocol was the same for the two study groups [30].

Blood samples were collected for AA concentrations on Whatman 903 grade filter paper at T0 (between three and seven days of life) and T1 (fifteen days of life) during neonatal metabolic screening. AA concentrations were determined via tandem mass spectrometry (ESI-MS/MS) using previously published methods [31] with some modifications. In brief, a 3-mm diameter dot was punched from a dried blood spot into a single well of 96-well micro plate. The dried blood spot was eluted in 100 μL of working extraction solution containing labelled internal standards. The sample was shaken for 30 min at 30 °C. Next, 65 μL of supernatant was dried under a nitrogen flow at 45 °C. The extracted AA were derivatized to butyl esters using 3 mol/l hydrochloric acid in *n*-butanol solution at 60 °C for 30 min. After derivatization, the sample was dried under a nitrogen flow at 45 °C and recovered using 50 μL of methanol/water (80:20) containing 0.1% acetic acid. Twenty microliters of the diluted sample were injected in flow injection analysis mode for the MS/MS experiments. Mobile phase was methanol/water (80:20) at a flow rate of 80 μL/min. AA signals were acquired using 102 amu neutral loss functions with the exceptions of ornithine, citrulline and arginine, whose signals were acquired in MRM mode. We collected data regarding arginine, citrulline, alanine, ornithine, leucine, isoleucine, proline, valine, glycine, methionine, phenylalanine and tyrosine. We also calculated the blood concentration of total AA, total essential (arginine, leucine, isoleucine, valine, methionine, phenylalanine and tyrosine) and total non-essential (citrulline, alanine, ornithine, proline and glycine) AA concentrations.

The levels of AA of 3500 at-term breastfed newborns, born in the enrollment period, were used as control reference values These values were derived from samples sent to our laboratory for the newborn screening at 48–72 h of life.

### 2.3. Statistical Analysis

Statistical analyses were performed using Statistical Package for Social Science software (SPSS Inc., Chicago, IL, USA), version 25.0. We determined variable normality using the Shapiro–Wilk test. The mean and 95% confidence interval summarized continuous variables, number variables and percentage category variables. We used the χ^2^ test for categorical variables and *t*-test or Mann–Whitney test for paired and unpaired variables. To evaluate the influence of covariates on blood AA values statistically significant in univariate analyses, we performed liner regression analyses, considering the value at T1 of blood AA as a dependent variable and the value at T0 of blood AA, non-protein energy intake and protein intake via PN, energy intake via EN, duration of PN, GA and BW as confounding variables. A *p*-value < 0.05 was considered significant for all statistical tests. The statistician was blind to the study aims and design.

## 3. Results

The baseline clinical characteristics and morbidity conditions during hospital stays are described in Table 1. No statistical differences were found between the two study cohorts (Table 1).

Cohort A received a statistically significant higher amount of total energy (total and non-protein), dextrose and lipid intake via PN in the first seven days of life compared to Cohort B, with the same protein intake (Table 2). The non-protein energy:protein intake via PN in the first week of life was statistically higher in Cohort A compared to Cohort B (Table 2). Dextrose:lipids intake via PN in the first week of life were lower in Cohort A compared to Cohort B (A 4.2, 95% CI 4.0–4.4 vs. B 4.6, 95% CI 4.2–5.0, *p* = 0.046). No differences were found for all macronutrient intakes via EN (human milk, preterm formula and both, Table 2).

**Figure 1:**
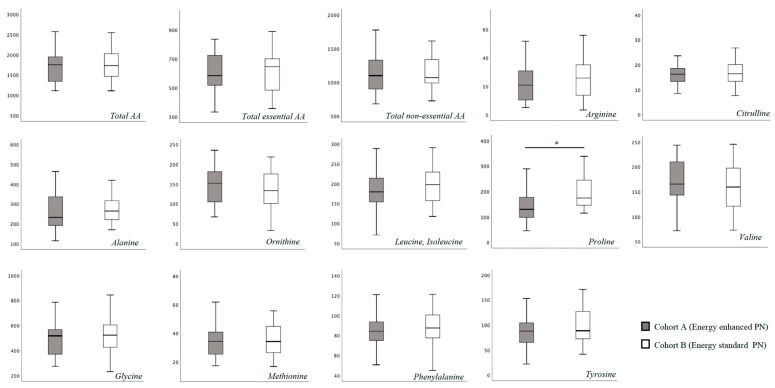
Plasma amino-acids concentration at T0 (between 3 and 7 days of life). Figure legend AA (Amino acids). All AA concentration were expressed as μmol/L. * *p* > 0.05.

**Figure 2:**
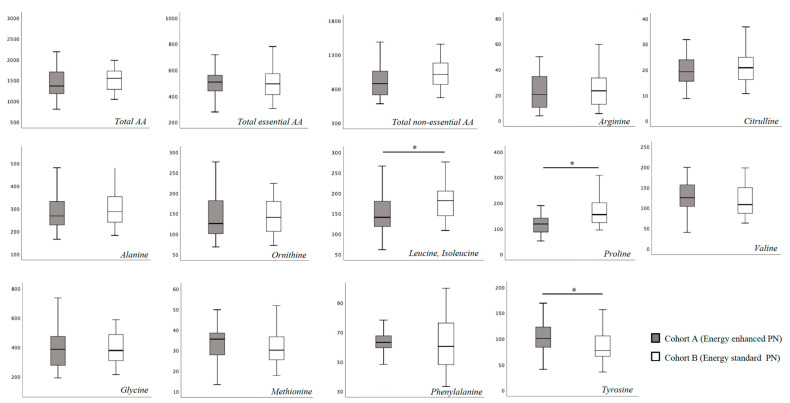
Plasma amino-acids concentration at 15 days of life. Figure legend AA (Amino acids). All AA concentration were expressed as μmol/L. * *p* > 0.05.

In Table S1 we report the blood AA levels of the study population, overall and according to cohort assignment. No substantial difference was found for total essential and non-essential blood AA concentration at T0 between the two study cohorts (Figure 1). Specifically, only proline showed a higher blood concentration in Cohort B compared to Cohort A (Figure 1). At 15 days of life, we found a significantly higher concentration of leucine, isoleucine and proline, and a significantly lower concentration of tyrosine in Cohort B (Figure 2).

**Figure 3:**
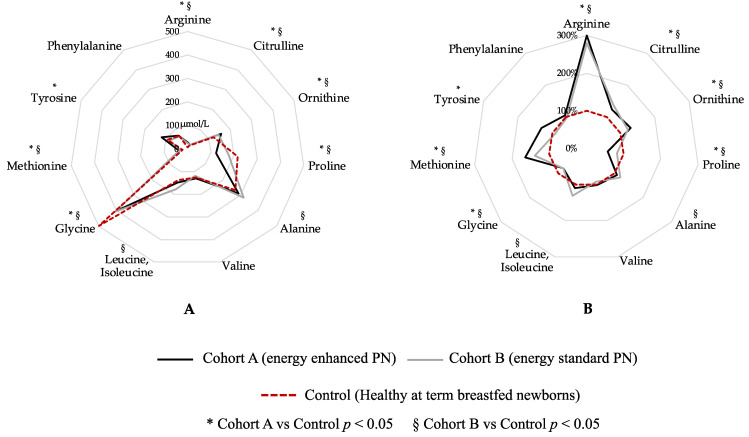
Graphical comparison of amino acid levels in the two study cohorts compared to healthy breastfed-at-term newborns. Figure legend: PN (parenteral nutrition). (**A**) Blood amino-acids expressed in µmol/L; (**B**) Blood amino-acid expressed in percentage, considering Control group (dashed red circle) as 100%.

Figure 3 shows a graphical comparison of AA levels in the two study cohorts at T1 compared with healthy, breastfed-at-term newborns (Figure 3). Blood levels of arginine, citrulline, ornithine, proline, glycine and methionine show a statistical difference between both Cohort A vs. Control and Cohort B vs. Control (Figure 3). Tyrosine shows a statistical difference between Cohort A and Control, while alanine and leucine, isoleucine shows a statistical difference between Cohort B and Control (Figure 3). Only valine and phenylalanine were similar between both Cohort A and Control and Cohort B and Control group (Figure 3).

Linear regression analysis showed a positive relationship between tyrosine levels at T1 and GA at birth (Table 3). No association was found between proline, leucine, isoleucine or tyrosine and other confounding variables at T1 (Table 3). The same model considering only nutritional support in the first 15 days of life confirmed the positive relationship between tyrosine levels at T1 and GA at birth. No association was found between proline, leucine, isoleucine, and other confounding variables in this multivariate model (Table 3).

## 4. Discussion

Early enhanced energy intake via PN did not influence blood AA levels and profiles in preterm newborns. In both study cohorts, PN protocol was defined according to actual recommendations and did not show satisfactory AA profiles compared to controls.

Previous studies demonstrated that AA supplementation via PN increases blood AA levels in preterm newborns, but did not investigate the role of energy intake [32,33]. Blanco et al. performed a randomized controlled trial (RCT) to evaluate the influence of two PN protocols, differing in terms of protein intake but not energy, on blood AA concentration at day one, three and seven of life [32]. The infants in the standard group received intravenous AA starting at 0.5 g/kg/d to a maximum of 3 g/kg/d, while infants in the early and high groups received 2 g/kg/d of intravenous AA soon after birth to 4 g/kg/d [32]. The researchers found a higher blood AA concentration in the latter group. In addition, AA concentration was detected at one, three and seven days of life; AA values evaluated so early after birth might still be a picture of fetal life status and not yet a consequence of PN. Finally, the influence of covariates on AA levels was not evaluated since a multivariate analysis was not performed. Clark et al. performed an RCT comparing two different groups of extremely preterm infants receiving two different PN protocols in terms of AA (starting dose 1.0 g/kg per day; target dose 2.5 g/kg per day vs. starting dose 1.5 g/kg per day; target dose 3.5 g/kg per day) with similar energy intake [33]. This study demonstrated that the dose of proteins in PN increases some blood AA concentrations in the first days of life (“parenteral phase of nutrition”) and at 28 days of life. However, these results were not adjusted for confounding variables.

Bulbul et al. performed an RCT to study the efficacy of early high doses via PN (in term of protein and fat) vs. low dose with progressive increments via PN regimens in VLBW infants [34]. Similar to our study, they found no differences at 14 days of life in the concentrations of AA except for arginine and asparagine, but they did not correct this result for confounding variables. In addition, Morgan et al. demonstrated that increasing early protein and energy intakes in very preterm infants did not prevent low blood levels of AA in the first 14 days of life, and there was an imbalance in essential AA provisions in the SCAMP trial [35]. However, contrasting our study, in the above-mentioned study there was also a difference in PN protein intakes, not only energy. Finally, unlike in this study, a multivariate analysis was not performed. Thus, it is not possible to establish, in Morgan et al., if AA levels depend on AA intake or on energy intake.

Our results suggest that an increased energy intake in early life may result in higher blood levels of some AA (i.e., leucine, isoleucine, alanine) and in a contemporary reduction of others (i.e., tyrosine) compared to the AA profile of breastfed full-term infants. The relationship between energy intake and blood AA levels in preterm newborns is still controversial. If some evidence suggested that increased energy intakes are associated with an increased anabolism with consequent AA consumption and reduced blood AA levels, other studies demonstrated that higher energy intakes led to increased levels of blood AA [36]. Despite reference values for blood AA in preterm newborns still being undefined, our study confirmed that PN prescriptions according to current guidelines results in non-optimal blood AA levels in preterm infants.

A key challenge in determining energy requirements is the interdependence of the energy fractions provided by the respective macronutrients. Some trials reported that AA supplementation in PN increases blood AA levels in preterm newborns, but did not investigate the role of energy intake with same protein support via PN and the energy:protein ratio. In enterally fed newborns, at equal protein and energy intakes, carbohydrates may result in higher nitrogen retention compared to fat, although this may be due to differences in absorption rates [5,37,38]. However, to the best of our knowledge, there are no studies with similar mechanisms for parenterally-fed ill newborns. Further studies are advocated to verify the relative proportion of macronutrients in the diet.

More recently, it has been demonstrated that high energy supply via PN could produce dangerous effects for VLBW. High energy supply via PN early in life increases the probability of metabolic complications, including hyperglycemia, that in turn reduce survival probability [9,16]. In addition, higher energy intake in the first week of life is associated with a poorer outcome in a long-term follow-up study [17]. Thus, considering that AA profiles are healthier in neonates receiving lower energy intake via PN, we believe that in the first week of life, high amounts of non-protein energy intakes should not be administered.

Despite these interesting findings, this study had some limitations. Our findings may be related to the effects of chance (random error), bias, or confounding factors. To limit this bias, we verified that the effects of AA concentration were influenced by confounding variables. We performed a linear regression analysis considering the PN of the first two weeks of life. There were no statistical differences between the two models. The only statistical relationship was between tyrosine levels at T1 and GA at birth, in both models. Despite our efforts, unknown confounding variables or those not considered in our statistical analysis may have influenced the study results. Indeed, AA metabolism is complex, and multiple factors could influence their blood level. Moreover, this is not a RCT. Individualized nutritional corrections are the milestone of our policy on PN, in order to avoid deleterious consequences of complications related to the administration of intravenous macronutrients [18]. Despite being a potential source of information bias, we have preferred that physicians taking care of babies were aware of the composition of PN, in order to make immediate corrections in the case of complications. In addition, the risk of lack of equipoise within neonatologists caring for preterm infants could be very high. On the other hand, the severity of clinical conditions may increase the use of PN. To limit selection bias, neonatologists evaluating eligibility used objective inclusion criteria (such as GA and BW), unaware of the aims of our study. A protocol for the collection, measurement, and interpretation of data was discussed and defined before beginning the study, but researchers not involved in clinical practice and eligibility assessment and who were unaware of the cohort assignment collected the data for the statistical analysis, and researchers that were unaware of the cohort assignment evaluated the blood AA concentrations. Despite no changes in the policies of care during the study period and the similar baseline characteristics of the two cohorts, it is not possible to exclude the possibility that unknown differences in the clinical practice or changes in the medical staff composition may have influenced the results. The number of enrolled newborns may limit the generalizability of the results. However, considering leucine, isoleucine, proline and tyrosine differences, we estimated a power of 83.7%, 96.8% and 41.1%, respectively, through post-hoc sample size calculation (0.05 of type one error, two-tailed test).

## 5. Conclusions

In conclusion, we have demonstrated for the first time that the administration of an energy enhanced PN protocol did not improve the AA profile of preterm newborns. In addition, our results suggest that the administration of PN according with current guidelines for preterm is not able to assure adequate blood AA profile. Together with the previously demonstrated increased risk of metabolic complications, such as hyperglycemia and hypertriglyceridemia, these results suggest exercising caution when administering high energy intake via PN in the first days of life.

## Figures and Tables

**Table 1:** Baseline characteristics and morbidity conditions of the study population.

	Cohort A (Energy Enhanced PN) *n* = 40	Cohort B (Energy Standard PN) *n* = 40
Gestational age, weeks	29.9 (29.2–30.7)	29.4 (28.7–30.2)
<29 weeks, No (%)	10 (25.0)	11 (27.5)
Birth weight, g	1262 (1163–1362)	1321 (1211–1430)
Extremely low birth weight, No (%)	6 (15.0)	6 (15.0)
Small for gestational age at birth, No. (%)	7 (17.5)	5 (12.5)
Male sex, No. (%)	22 (55.0)	27 (67.5)
Cesarean section, No. (%)	37 (92.5)	35 (87.5)
Antenatal corticosteroids a, No. (%)	29 (72.5)	30 (75.0)
Intrauterine growth restriction, No (%)	3 (7.5)	3 (7.5)
Twins, No. (%)	7 (17.5)	13 (32.5)
5-min Apgar score	8 (7–8)	8 (7–8)
pH at birth	7.26 (7.23–7.29)	7.25 (7.22–7.29)
CRIB II score	6 (5–7)	6 (5–8)
Start of EN, days of life	2 (1–3)	1 (1–2)
Start of EN before to 72 h, No (%)	34 (85.0)	36 (90.0)
FEF before 7 days of life, No (%)	13 (32.5)	13 (32.5)
Duration of PN, days	13 (9–17)	12 (9–15)
PN more than 70% 0–7 DOL, No (%)	18 (45.0)	23 (57.5)
Necrotizing enterocolitis (Bell Stage ≥ 3), No (%)	1 (2.5)	0 (0)
Intraventricular hemorrhage, No (%)	1 (2.5)	4 (10.0)
Periventricular leukomalacia, No (%)	1 (2.5)	1 (2.5)
Sepsis proven by positive culture, No (%)	3 (7.5)	3 (7.5)
Retinopathy of prematurity (Stage ≥ 2), No (%)	3 (7.5)	5 (12.5)
Bronchopulmonary dysplasia, No (%)	2 (5.0)	3 (7.5)
Overall Morbidity b, No (%)	10 (25.0)	9 (22.5)
Respiratory support, No (%)	30 (75.0)	31 (77.5)
Survival, No (%)	39 (97.5)	39 (97.5)
Length of hospital stay, days	59 (48–70)	57 (48–67)

Table legend. Data are shown as mean (95% confidence interval), where not specified; (a) Intramuscular steroid cycle in two doses of 12 mg over a 24-h period; (b) at least one of the prematurity-related morbidity conditions mentioned above; CRIB (Clinical risk index for babies); EN (Enteral nutrition); FEF (Full enteral feeding); PN (Parenteral nutrition); DOL (Days of life).

**Table 2:** Macronutrients received in the first week of life by the study population.

	Cohort A (Energy Enhanced PN) *n* = 40	Cohort B (Energy Standard PN) *n* = 40
Total energy intake via PN in the first week of life (kcal/kg/week)	485.0 (416.6–553.5) *	326.9 (260.3–393.6)
Non-protein energy intake via PN in the first week of life (kcal/kg/week)	418.2 (359.8–476.5) *	275.6 (220.4–330.8)
Protein intake via PN in the first week of life (g/kg/week)	16.7 (14.1–19.3)	12.8 (9.9–15.8)
Dextrose intake via PN in the first week of life (g/kg/week)	66.1 (57.3–74.8) *	44.5 (35.6–53.4)
Lipids intake via PN in the first week of life (g/kg/week)	16.3 (13.7–18.8) *	10.5 (8.3–12.7)
Non-protein energy intake: Protein intake via PN in the first week of life	25.4 (24.9–25.9) *	22.7 (21.7–23.8)
Energy intake via EN (HM + PF) in the first week of life (kcal/kg/week)	178.1 (122.5–233.7)	183.4 (129.9–236.8)
Protein intake via EN (HM + PF) in the first week of life (g/kg/week)	6.0 (4.1–7.9)	6.1 (4.3–7.9)
Dextrose intake via EN (HM + PF) in the first week of life (g/kg/week)	18.2 (12.5–23.9)	18.6 (13.2–24.1)
Lipids intake via EN (HM + PF) in the first week of life (g/kg/week)	9.1 (6.2–11.9)	9.4 (6.7–12.2)
Energy intake via HM in the first week of life (kcal/kg/week)	55.0 (27.5–82.4)	62.7 (30.1–95.3)
Protein intake via HM in the first week of life (g/kg/week)	1.5 (0.7–2.2)	1.7 (0.8–2.6)
Dextrose intake via HM in the first week of life (g/kg/week)	5.3 (2.7–8.0)	6.1 (2.9–9.2)
Lipids intake via HM in the first week of life (g/kg/week)	3.1 (1.6–4.7)	3.6 (1.7–5.4)
Energy intake via PF in the first week of life (kcal/kg/week)	135.5 (835–187.5)	130.1 (86.6–173.6)
Protein intake via PF in the first week of life (g/kg/week)	4.9 (3.0–6.7)	4.7 (3.1–6.2)
Dextrose intake via PF in the first week of life (g/kg/week)	14.1 (8.7–19.4)	13.5 (9.0–18.0)
Lipids intake via PF in the first week of life (g/kg/week)	6.7 (4.1–9.3)	6.4 (4.3–8.6)

Table legend. Data are shown as mean (95% confidence interval); PN (Parenteral nutrition); EN (Enteral nutrition); HM (Human milk); PF (Preterm Formula); * *p* < 0.05 vs. Cohort B.

**Table 3:** Linear regression analysis to evaluate the influence of covariates on plasma amino-acids concentration at T1 (15 days of life).

	Variables	B	S.E.	ß	*p* Value	95 CI for OR	
						Lower	Upper
**Leucine, Isoleucine** **(T1)**	Leucine, Isoleucine (T0)	0.224	0.146	0.241	0.132	−0.070	0.518
	Non-protein energy intake via PN in the first week of life	−0.112	0.161	−0.415	0.491	−0.435	0.212
	Protein intake via PN in the first week of life	−1.539	4.175	−0.248	0.714	−9.948	6.870
	Energy intake via EN in the first two weeks of life	−0.028	0.029	−0.282	0.328	−0.086	0.029
	Duration of PN	0.887	1.191	0.186	0.460	−1.511	3.285
	Gestational age	3.566	5.307	0.141	0.505	−7.122	14.254
	Birth weight	−0.059	0.038	−0.351	0.125	−0.135	0.017
**Proline** **(T1)**	Proline (T0)	0.199	0.167	0.190	0.238	−0.137	0.535
	Non-protein energy intake via PN in the first week of life	−0.161	0.253	−0.386	0.529	−0.671	0.350
	Protein intake via PN in the first week of life	−2.532	6.367	−0.264	0.693	−15.355	10.291
	Energy intake via EN in the first two weeks of life	−0.083	0.046	−0.536	0.076	−0.176	0.009
	Duration of PN	−1.053	1.880	−0.142	0.578	−4.840	2.734
	Gestational age	0.769	7.958	0.020	0.923	−15.259	16.796
	Birth weight	−0.113	0.059	−0.433	0.063	−0.232	0.006
**Tyrosine** **(T1)**	Tyrosine (T0)	−0.047	0.138	−0.049	0.737	−0.325	0.232
	Non-protein energy intake via PN in the first week of life	0.118	0.347	0.197	0.736	−0.580	0.816
	Protein intake via PN in the first week of life	0.386	8.981	0.028	0.966	−17.702	18.475
	Energy intake via EN in the first two weeks of life	0.068	0.061	0.304	0.274	−0.056	0.192
	Duration of PN	1.535	2.627	0.145	0.562	−3.756	6.826
	Gestational age	25.956	12.126	0.462	0.038	1.533	50.379
	Birth weight	−0.102	0.088	−0.274	0.253	−0.280	0.075
**Leucine, Isoleucine** **(T1)**	Leucine, Isoleucine (T0)	0.202	0.149	0.217	0.183	−0.099	0.502
	Non-protein energy intake via PN in the first two weeks of life	−0.097	0.113	−0.851	0.392	−0.324	0.130
	Protein intake via PN in the first two weeks of life	1.457	2.808	0.552	0.606	−4.199	7.112
	Energy intake via EN in the first two weeks of life	−0.013	0.037	−0.129	0.726	−0.087	0.061
	Duration of PN	1.184	1.255	0.248	0.350	−1.344	3.713
	Gestational age	4.813	5.503	0.190	0.386	−6.271	15.898
	Birth weight	−0.021	0.043	−0.123	0.629	−0.106	0.065
**Proline** **(T1)**	Proline (T0)	0.210	0.170	0.200	0.224	−0.133	0.553
	Non-protein energy intake via PN in the first two weeks of life	−0.157	0.177	−0.883	0.380	−0.512	0.199
	Protein intake via PN in the first two weeks of life	1.696	4.304	0.415	0.695	−6.972	10.364
	Energy intake via EN in the first two weeks of life	−0.074	0.058	−0.477	0.209	−0.192	0.043
	Duration of PN	−0.430	1.937	−0.058	0.825	−4.330	3.471
	Gestational age	2.443	8.214	0.062	0.768	−14.101	18.987
	Birth weight	−0.074	0.065	−0.283	0.266	−0.206	0.058
**Tyrosine** **(T1)**	Tyrosine (T0)	−0.047	0.141	−0.049	0.743	−0.332	0.238
	Non-protein energy intake via PN in the first two weeks of life	0.041	0.241	0.161	0.866	−0.445	0.527
	Protein intake via PN in the first two weeks of life	−0.595	5.986	−0.102	0.921	−12.651	11.461
	Energy intake via EN in the first two weeks of life	0.048	0.076	0.215	0.531	−0.105	0.201
	Duration of PN	1.304	2.648	0.123	0.625	−4.030	6.639
	Gestational age	25.756	12.200	0.458	0.040	1.183	50.329
	Birth weight	−0.139	0.096	−0.371	0.155	−0.332	0.054

Table legend. CI (confidence interval); T0 (between 3 and 7 days of life); T1 (15 days of life); PN (Parenteral nutrition); EN (Enteral nutrition).

## Data Availability

All data relevant to the study are included in the manuscript. Data will be available to any researcher from the corresponding author upon reasonable request.

## Supplementary Materials

The following supporting information can be downloaded at: https://www.mdpi.com/article/10.3390/nu15132917/s1, Table S1: Blood amino acids concentration of the study population.
