# Supplementary material for: Early Energy Intake and Amino Acid Profile in Preterm Newborns: A Quasi-Experimental Study
Source: Nutrients. 2023 Jun 27;15(13):2917. doi: 10.3390/nu15132917 (PMC10343583; doi:10.3390/nu15132917)
Supplement: Supplementary file 1 [file nutrients-15-02917-s001.zip › nutrients-2448804-supplementary.pdf]

Table S1. Blood amino acids concentration of the study population.

		Total preterm newborn (n=80)	Cohort A (Energy enhanced PN) (n=40)	Cohort B (Energy standard PN) (n=40)
T0	Total AA, µmol/L	1722.8 (1632.6-1813.1)	1719.5 (1569.9-1878.2)	1763.8 (1632.8-1894.8)
	Total essential AA, µmol/L	615.5 (578.4-652.4)	607.4 (557.5-657.3)	625.9 (558.7-693.3)
	Total non-essential AA, µmol/L	1107.4 (1041.4-1173.0)	1112.1 (884.7-1229.6)	1137.8 (1050.9-1224.7)
	Arginine, µmol/L	26.2 (21.9-30.4)	24.7 (18.9-30.6)	27.7 (21.3-34.1)
	Citrulline, µmol/L	17.1 (15.7-18.5)	16.3 (14.8-17.7)	18.0 (15.5-20.5)
	Alanine, µmol/L	268.7 (248.6-288.9)	263.1 (228.5-297.8)	274.5 (252.6-296.4)
	Ornithine, µmol/L	143.0 (131.7-154.3)	149.1 (133.1-165.1)	136.7 (120.1-153.3)
	Leucine, Isoleucine, µmol/L	189.4 (178.0-200.8)	182.0 (165.9-198.2)	196.9 (180.5-213.4)
	Proline, µmol/L	170.8 (155.3-186.3)	147.7 (126.8-168.7) *	194.5 (173.5-215.5)
	Valine, µmol/L	166.3 (155.7-176.8)	170.5 (156.4-184.6)	161.9 (145.6-178.2)
	Glycine, µmol/L	507.8 (474.2-541.3)	496.0 (444.7-547.3)	519.9 (474.5-565.2)
	Methionine, µmol/L	36.5 (32.1-40.8)	34.3 (30.3-38.3)	38.7 (30.6-46.8)
	Phenylalanine, µmol/L	87.4 (83.4-91.4)	87.2 (81.3-93.2)	87.5 (81.8-93.3)
	Tyrosine, µmol/L	109.6 (86.8-132.5)	96.1 (77.3-114.8)	123.6 (80.7-166.5)
T1	Total AA, µmol/L	1550.8 (1440.2-1661.5)	1472.9 (1294.9-1650.9)	1580.3 (1419.7-1741.0)
	Total essential AA, µmol/L	518.8 (479.8-557.8)	519.0 (445.7-592.4)	502.3 (462.3-542.4)
	Total non-essential AA, µmol/L	1031.9 (946.2-1117.7)	953.9 (824.9-1082.9)	1078.0 (942.1-1213.9)
	Arginine, µmol/L	27.3 (21.2-33.3)	28.3 (16.7-39.8)	26.4 (20.1-32.6)
	Citrulline, µmol/L	20.7 (19.1-22.3)	19.7 (17.5-21.9)	21.5 (19.2-23.8)
	Alanine, µmol/L	303.2 (275.6-330.8)	287.9 (252.3-323.4)	316.1 (273.9-358.2)
	Ornithine, µmol/L	150.4 (134.2-166.7)	155.9 (125.6-186.1)	145.9 (128.5-163.3)
	Leucine, Isoleucine, µmol/L	165.6 (154.3-176.8)	149.9 (132.9-166.7) *	178.7 (164.3-193.1)
	Proline, µmol/L	152.6 (135.9-169.3)	123.9 (107.1-140.9) *	176.6 (151.4-201.7)
	Valine, µmol/L	123.5 (113.1-133.8)	130.3 (112.9-147.6)	117.8 (105.0-130.6)
	Glycine, µmol/L	404.9 (363.9-445.9)	403.9 (340.8-467.1)	405.8 (349.3-462.3)
	Methionine, µmol/L	33.4 (29.6-37.1)	36.5 (28.8-44.2)	30.8 (28.1-33.5)
	Phenylalanine, µmol/L	66.5 (60.7-72.3)	70.2 (60.7-79.7)	63.5 (56.1-70.8)
	Tyrosine, µmol/L	102.6 (80.2-125.0)	122.5 (74.1-170.9) *	86.0 (76.1-95.9)

Table legend. Data are shown as mean (95% confidence interval); T0 (between 3 and 7 days of life); T1 (at 15 days of life); * p value < 0.05 vs. Cohort B.
